# Functional constipation in pediatric patients: an observational study in southern Brazil

**DOI:** 10.1590/1516-3180.2025.2853.13062025

**Published:** 2025-09-01

**Authors:** Anita dos Santos Cardoso, Laura Bittencourt de Oliveira, Mayra Sonego

**Affiliations:** I Medical Student, Department of Health Sciences, Universidade do Extremo Sul Catarinense (Unesc), Criciúma (SC), Brazil.; II Medical Student, Department of Health Sciences, Universidade do Extremo Sul Catarinense (Unesc), Criciúma (SC), Brazil.; III Pediatrician; Professor, Department of Health Sciences, Universidade do Extremo Sul Catarinense (Unesc), Criciúma (SC), Brazil.

**Keywords:** Constipation., Pediatrics., Gastrointestinal diseases., Constipation, Pediatric prevalence, Gastrointestinal disorders, Abdominal pain, Stool retention

## Abstract

**BACKGROUND:**

Functional constipation is characterized by a set of symptoms including hardened stools, abdominal discomfort, a tendency to retain stools, and eventual fecal incontinence. This condition negatively affects the quality of life of the affected individuals and has potential psychosocial repercussions.

**OBJECTIVES:**

To assess the epidemiological, clinical, and therapeutic aspects of functional constipation in patients treated at a pediatric gastroenterology outpatient clinic.

**DESIGN AND SETTING:**

Descriptive observational study with a quantitative approach using secondary data collected from the medical records of a pediatric gastroenterology outpatient clinic in Criciúma between 2018 and 2023.

**METHODS:**

This study was approved by the Human Research Ethics Committee of the Universidade do Extremo Sul Catarinense (Unesc) under number 6.788.465. Sociodemographic, clinical, and therapeutic variables were evaluated in 67 patients aged 0–18 years who were diagnosed with Functional Constipation (ICD K590) during the study period. Data were analyzed using descriptive statistics in the Statistical Package for the Social Sciences (SPSS), version 25.0.

**RESULTS:**

There was a predominantly female profile (36; 53.7%), with an average age at diagnosis of 7.75 years (± 3.96). The main symptoms included abdominal pain (52, 77.6%) and hardened stools (42, 62.7%), with an average interval of 4 days between bowel movements. Treatment consisted of macrogol prescriptions (60, 89.6%), with most patients showing complete symptom improvement (49, 73.1%).

**CONCLUSION:**

Analysis of medical records highlighted the need for continuous monitoring and targeted interventions, considering the variability of symptoms and individual characteristics of patients.

## INTRODUCTION

 Functional gastrointestinal disorders are a group of medical conditions characterized by the persistence or recurrence of gastrointestinal symptoms, even in the absence of identifiable structural or biochemical alterations.^1^ A prominent subtype is functional constipation (FC), which warrants specific attention.^2^

 FC presents as persistent difficulty with bowel movements, which may involve excessive straining, hardened stools, and occasionally, fecal incontinence.^3^ This condition, diagnosed by the Rome IV criteria,^3^ has a significant impact on patients’ quality of life, leading to pronounced abdominal discomfort and psychosocial repercussions.^1^

 The global epidemiology of pediatric constipation has an estimated prevalence of 9.5%, with notable variations across regions, ranging from 0.5% to 32.2%.^4^ In the Brazilian context, the latest data on prevalence indicated rates between 17.5% and 36.5%, suggesting a higher number of affected individuals compared to the global average.^5^

 Biopsychosocial risk factors such as stress, childhood obesity, sedentary lifestyle, poor diet, and dysfunctional family environments may exacerbate symptoms.^6^ Therefore, treatment aims at targeted interventions for these aspects, necessitating changes in dietary patterns, lifestyle, and medication use.^ 6,7^ Macrogol, an osmotic laxative, is the primary substance used in the management of functional constipation across all age groups because of its efficacy and safety in long-term treatment.^7^

 FC in children and adolescents is often underestimated and can substantially affect their quality of life, affecting daily activities and socio-emotional aspects. Characterizing pediatric patients with FC provides a foundation for identifying specific patterns and enabling more efficient and targeted treatments. This significantly improves clinical practice and the well-being of these patients, leading to positive long-term outcomes. This study aimed to describe the epidemiological, clinical, and therapeutic aspects of patients with FC treated at the pediatric gastroenterology department of the Secretaria Municipal de Saúde de Criciúma (PMC) between 2018 and 2023. 

## METHODS

### Study design

 This was a descriptive observational study with secondary data collection. 

### Ethical aspects

 This study was approved by the Research Ethics Committee of the Universidade do Extremo Sul Catarinense (Unesc) under number 6.788.465. The requirement for informed consent was waived because this was a retrospective study based on medical records with no identification of the participants. 

### Population

 The target population included all individuals aged 0–18 years diagnosed with functional constipation (ICD K590), who were treated at a specialized pediatric gastroenterology service in the southern region of Santa Catarina between January 2018 and December 2023. Patients with an inconclusive diagnosis of functional constipation according to the Rome IV criteria were excluded. 

### Sample

 The medical records of all patients fitting the target population during the period of January 2018 to December 2023 were included in the study, considering it a census data collection.^8^ A total of 71 medical records were obtained during the evaluated period. 

### Data collection

 Data from patients aged 0–18 years who were diagnosed with functional constipation (ICD K590) and treated at the pediatric gastroenterology outpatient clinic between 2018 and 2023 were analyzed. Medical records were retrieved from the Celk Saúde system in the Municipality of Criciúma. The variables analyzed included patient age, sex, age at symptom onset, signs and symptoms associated with functional constipation, interval between bowel movements, fecal incontinence episodes, nutritional quality, presence of comorbidities, daily medication use, prior macrogol treatment and dose, prescribed treatment, and clinical response. 

 The data collection instrument was a questionnaire developed by the researchers structured into four distinct sections to cover the research variables and provide a detailed understanding of the clinical and therapeutic panorama of children treated in the pediatric gastroenterology service. Section A contains the essential information for patient identification and characterization, including medical record number, age, and sex. Section B (Past Medical History) focused on evaluating the patients’ medical history and gathering information on comorbidities and medication use. Section C explores the clinical details related to functional constipation and investigates factors such as abdominal pain and fecal incontinence. Section D addresses information on therapeutic interventions for gastrointestinal conditions as well as the observed clinical responses. 

### Statistical analysis

 Data were analyzed using IBM Statistical Package for the Social Sciences (SPSS), version 25.0. Descriptive analyses of the studied variables were performed, reporting the frequency and percentage of qualitative variables (sex, daily medications, comorbidities, nutritional quality, signs and symptoms, presence of fecal incontinence, prior treatment, type of treatment, dietary changes, and clinical response) and the mean and standard deviation of quantitative variables (current age, age at symptom onset, interval between bowel movements, and prior macrogol dose) if they followed a normal distribution, and median and interquartile range if they did not. The normality of the quantitative variables was assessed using the Kolmogorov-Smirnov test. All the results are expressed as graphs and/or tables. 

## RESULTS

 During the analysis period, 71 medical records of patients aged 0–18 years with functional constipation (ICD K590) were selected. After excluding four cases with inconclusive diagnoses, the final sample comprised 67 patients, corresponding to 9.4% of the total (710) individuals who attended the specialized outpatient clinic. The mean age at the time of consultation was 7.75 (± 3.96) years. The minimum and maximum ages ranged from 1–16 years, with a predominance of females (36; 53.7%) (**Table 1**). 

**Table 1:** Demographic characteristics of patients with functional constipation

**Variables**		**Mean ± SD, n (%)**
		**n = 67**
Age (years)		7.75 ± 3.96
	*Minimum*	1
	*Maximum*	16
Sexo		
	*Female*	36 (53.7)
	*Male*	31 (46.3)

 The average age of onset of functional constipation symptoms was 4.49 (± 3.93) years, with a predominance in younger age groups, as most episodes occurred in children up to 5 years old (41; 66.1%). The interval between the onset of the first symptoms and specialized consultation was at most two years in the majority of cases (32; 47.8%). 

 Among the signs and symptoms, the most prevalent were abdominal pain (52; 77.6%), which was particularly common in patients up to 10 years of age (43; 82.7%); hardened stools (42; 62.7%); and hematochezia (28; 41.8%), which were more common in the 6–15 years age group (21; 75%). Other manifestations such as excessive straining (20, 29.9%), abdominal distension (16, 23.9%), and fecal impaction (16, 23.9%) appeared in approximately one-third of the patients (**Table 2**). 

**Table 2:** Clinical characteristics of patients with functional constipation

**Variables**			**Mean ± SD, n (%)**
			**n = 67**
**Age of symptom onset**			**4.49 ± 3.93**
	Signs and symptoms		
		*Abdominal pain*	52 (77.6)
		*Hardened stools*	42 (62.7)
		*Hematochezia*	28 (41.8)
		*Excessive straining*	20 (29.9)
		*Abdominal distension*	16 (23.9)
		*Impaction and/or rectal Mass*	16 (23.9)
		*Fear of defecation*	14 (20.9)
		*Nausea and/or vomiting*	10 (14.9)
		*Tenesmus*	10 (14.9)
		*Abdominal colic*	4 (6)
	Interval between bowel movements (days)		
		*1–3 days*	16 (23.9)
		*4–10 days*	33 (49.3)
		*14–30 days*	5 (7.5)
		*Ignored*	13 (19.4)
	Fecal incontinence		
		*Yes*	23 (34.3)
		*No*	44 (64.7)
	Dietary quality		
		*Varied diet*	41 (61.2)
		*Selective diet*	24 (35.8)
		*Ignored*	2 (3)

 The interval between bowel movements varied, ranging from a minimum of 1 (4; 6%) to a maximum of 30 days (1; 1.5%). However, the most common interval was 4 days (n = 15; 22.4%). Constipated patients with longer periods between bowel movements (4 or more days) had a higher occurrence of fecal incontinence episodes (15; 83.3%). In total, 23 (34.3%) reported this symptom. Despite dietary habits, 61.2% (n = 41) of patients followed a varied diet, while 35.8% (n = 24) ateselectively e. The occurrence rates of up to four symptoms were similar in both cases at 87% (36) and 83.3% (20). 

 Comorbidities were present in 34.9% (23) of the patients and were more prevalent in boys (14; 60.9%). A minority of patients were on daily medication (18, 26.9%). **Table 3** shows the clinical characteristics of the patients. The main coexisting conditions among those with functional constipation included Attention Deficit Hyperactivity Disorder (ADHD), Autism Spectrum Disorder (ASD), and Intellectual Disability, each affecting six patients (26.1%). Of the 18 individuals using medications, risperidone was the most commonly used (10; 55.6%), primarily in the 6 to 15 years age group. 

**Table 3:** Clinical history of patients with functional constipation

**Variables**			**Mean ± SD, n (%)**
			**n = 67**
**Comorbidities**			
	Yes		23 (34.9)
		*Attention deficit hyperactivity disorder*	6 (26.1)
		*Autism spectrum disorder*	6 (26.1)
		*Intellectual disability*	6 (26.1)
		*Cerebral palsy*	2 (8.7)
		*Oppositional defiant disorder*	2 (8.7)
		*Down syndrome*	1 (4.3)
		*Others*	6 (26.1)
	No		44 (65.7)
**Medication use**			
	Yes		18 (26.9)
		*Risperidone*	10 (55.6)
		*Imipramine*	7 (38.9)
		*Methylphenidate*	4 (22.2)
		*Amitriptyline*	2 (11.1)
		*Sodium valproate*	2 (11.1)
		*Fluoxetine*	1 (5.6)
		*Others*	9 (50)
	No		49 (73.1)

 Among the individuals studied, a minority (24; 35.8%) had previously used macrogol, mainly at lower doses (11; 45.8%), and in only one patient (0.04%), at a higher dose than that prescribed by the specialist. The average previous dose, excluding records without explicit registration (12, 50%), was 13.99 g ± 8.25. Regarding prescribed pharmacological treatment, only seven children (10.4%) did not receive a prescription for macrogol, making it the main recommended medication (60; 89.6%). Other medications included lactulose and enemas (both in 17 patients; 25.4%), sorbitol + sodium lauryl sulfate (8 patients; 11.9%), and mineral oil (7 patients; 10.4%). **Table 4** shows the details of the observed therapies. 

**Table 4:** Therapeutic resources used in patients with functional constipation

**Variables**			**Mean ± SD, n (%)**
			**n = 67**
**Previous treatment with macrogol**			
	Yes		24 (35.8)
		*Higher dose*	1 (0.04)
		*Lower dose*	11 (45.8)
		*Ignored*	12 (50)
	No		43 (64.2)
**Previous dose of macrogol**			13.99 ± 8.25
**Pharmacological treatment**			
	Prescribed medications		
		*Macrogol*	60 (89.6)
		*Lactulose*	17 (25.4)
		*Enema*	17 (25.4)
		*Sorbitol + sodium lauryl sulfate*	8 (11.9)
		*Mineral oil*	7 (10.4)
		*Macrogol 3350 + sodium bicarbonate + sodium chloride + potassium chloride*	1 (1.5)
		*Others*	3 (4.5)
	Number of medications prescribed per patient		
		*Up to 1 medication*	29 (59.2)
		*2–3 medications*	16 (32.7)
		*4 or more medications*	4 (8.2)
**Non-pharmacological treatment**			
	Dietary modifications		38 (56.7)
		*Increased fiber intake*	15 (39.5)
		*Remove cow’s milk*	8 (21.1)
		*Remove wheat*	1 (2.6)
		*Increased fluid intake*	31 (81.6)
	Lifestyle change		35 (52.2)
**Clinical response**			
	Complete improvement		49 (73.1)
	Partial improvement		8 (11.9)
	No change		1 (1.5)
	Worsening		2 (3)
	Relapse		1 (1.5)
	Did not return for follow-up		6 (9)

 Nonpharmacological treatments have also been adopted in addition to drug interventions. Dietary modifications were recommended for just over half of the patients (38; 56.7%), with increased fiber intake and water consumption being the main suggestions, as indicated for 15 (39.5%) and 31 (81.6%) patients, respectively. Lifestyle changes, such as increased physical activity and the establishment of regular evacuation routines, were proposed for 35 (52.2%) individuals. Isolated non-drug therapy was prescribed for only three (4.47%) constipated patients, highlighting the need for combined interventions to restore the intestinal health of those evaluated. 

 Regarding the clinical response, in the majority of the sample, complete symptom improvement was achieved with the use of up to one medication (29; 59.2%), which was the outcome observed in most patients (49; 73.1%). However, eight individuals (11.9%) showed partial improvement, and two (3%) reported worsening of symptoms. Additionally, six patients (9%) did not return for follow-up, limiting the reevaluation and prognosis of this group. 

## DISCUSSION

 In the pediatric gastroenterology outpatient clinic, functional constipation was predominantly observed in girls, with an average age of 7.75 years at the time of consultation and initial episodes occurring mostly before the age of 5 years. The main reported symptoms were abdominal pain and hardened stools, with an average interval of 4 days between bowel movements. Additionally, 34.3% of the patients experienced episodes of fecal incontinence. 

 The overall prevalence of functional constipation in the population, including both adults and children, shows wide variation, with estimates ranging from 0.7% to 79% and a median of 16%.^9^ In children, the rates range from 9.5% to 11.8%,^10,11^ with reports from specialized pediatric outpatient clinics in Brazil ranging from 7.6% to 29%.^12,13^ Internationally, the variation ranges from 3% to 46.7%, reflecting methodological and socioeconomic differences among studies.^14 ,15^ In this study, the prevalence of consultations for functional constipation of 9.4%, was within the expected range for both the national and global pediatric population. This result supports previously published data, suggesting that, even in a regional sample, the observed rates remain consistent with those found in other locations. 

 The mean age at the time of the consultation was 7.75 years (± 3.96), ranging from 1 to 16 years. Although the age group with the highest prevalence of functional constipation has not been clearly defined,^14^ the data obtained are similar to those reported in other studies, which reported average ages of 5.4 and 6.4 years.^16,9^ These results suggest that functional constipation can occur at various pediatric ages, without a specific age trend. Regarding sex, there was a female predominance (36; 53.7%), which aligns with findings showing similar prevalence rates between boys and girls (8.6% versus 8.9%, respectively).^4^ A Colombian study also found no significant gender predominance,^17^ indicating that the differences observed in our sample may be specific to the analyzed population. 

 The data presented reveal the onset of symptoms of FC at ages younger than 5 years, with an age of 4.49 years (± 3.93). The predominance of initial episodes in children up to 5 years old, representing 66.1% of the cases, is consistent with existing literature, which predicts an average onset age around 2.3 years, with higher prevalence in preschool-aged children.^5 ,18-20^ This suggests that factors such as dietary changes, transitions in sphincter control, and the start of schooling may play a significant role in triggering symptoms in this age group.^6,21-23^ It was also observed that 47.8% of individuals waited up to 2 years to receive specialized care, reflecting a pattern consistent with studies that reported that 77.8% of patients experience delays of over a year for their first consultation.^13,24^ This delay in treatment can exacerbate symptoms and lead to excessive use of rectal laxatives and other inappropriate interventions, resulting in significant morbidity for the pediatric population.^25^

 Hard stools are the most prevalent symptom of pediatric functional constipation, affecting nearly all patients.^5,19,26^ In the present sample, this symptom was observed in 62.7% of the cases, a notable rate but lower than that reported in other Brazilian studies, which indicated prevalence of up to 86.5%.^13^ This difference may suggest that some patients already experience partial symptom relief, possibly due to prior laxative use, or that the studied population has distinct clinical characteristics that influence this manifestation. On the other hand, abdominal pain was reported by 77.6% of the patients, a rate significantly higher than the 40% found in comparative studies.^27^ This symptom can significantly contribute to children’s resistance to evacuation, fueling a cycle of pain and retention that exacerbates constipation condition.^28-30^

 Most individuals experienced fewer than two bowel movements per week. Additionally, children with FC and longer intervals between evacuations (4 or more days) showed a higher incidence of fecal incontinence (15; 83.3%), reflecting the significant overlap between these conditions, which is often observed in more severe cases.^28,31 ,32^ Patients who experience this co-occurrence have a lower quality of life than those with isolated constipation,^24,33^ and if not properly treated, fecal incontinence can lead to social isolation and long-term psychological issues.^24^ Studies suggest that about one-quarter of patients experience this co-occurrence,continue to have symptoms in adulthood,^34^ and present a lower quality of life than those with worse clinical outcomes over time.^31^ This underscores the importance of early diagnosis and appropriate treatment. FI can lead to social isolation and long-term psychological issues.^7^

 Regarding diet, although some studies have shown associated inadequate eating habits, such as low fiber intake and functional constipation,^1,3,35^ others have found no significant correlation between diet and the disease.^32 ,36^ These findings are consistent with those of this study, where most patients followed a varied diet (41; 61.2%), suggesting that dietary factors may play a less determining role in some pediatric populations. However, it is crucial to consider dietary interventions as part of a multidisciplinary approach for managing functional constipation.^7,37^

 It was observed that 34.9% of the patients had comorbidities mainly characterized by neurodevelopmental disorders. The prevalence of ADHD in this sample (26.1%) was higher than the average reported in the literature (13.87 %),^38^ suggesting particularities of the study population. However, this comorbidity is not necessarily associated with a higher risk of gastrointestinal disorders compared to the general population, and medication for ADHD does not seem to significantly affect the rates of consultations for defecatory disorders.^38,39^ In contrast, children with ASD have a high prevalence of constipation, occurring in up to 85% of cases,^26^ and also a higher risk of needing medication, as exemplified by the use of risperidone in 26.9% of patients in this study. Constipation is a common side effect of risperidone in children with ASD,^40,41^ suggesting a possible relationship between the use of antipsychotics and the worsening of gastrointestinal symptoms. 

 In the literature, most patients received prior treatment in primary care before being referred to a specialist, with the main interventions being the use of laxatives (such as macrogol), dietary modifications, and lifestyle changes.^18,42^ Malowitz et al.^18^ and Bongers et al.^34^ found that although the peak onset of functional constipation occurs early, the search for specialized care usually occurs later, after prolonged episodes and inadequate management in primary care, with the presentation of more severe symptoms, indicating that previous treatments were insufficient.^18,34^ In the present study, we found a minority of individuals (24; 35.8%) who had used macrogol previously, with an estimated average dose of 13.99 g ± 8.25. Although this average does not take into account the children’s weight, and thus it is not possible to confirm whether it is within the recommended dose range of 0.93 ± 0.28 g/kg/day, with a maximum of 30 g/day,^43^ it is plausible that the previous treatment followed guidelines, even though the available data do not allow for a conclusive analysis. 

 Macrogol is the primary pharmacological treatment for pediatric functional constipation, as recommended by the guidelines of the North American Society for Pediatric Gastroenterology, Hepatology, and Nutrition, due to its safety and efficacy in increasing the water content in stools, thus relieving symptoms.^44,45^ When it is unavailable or poorly tolerated, lactulose is the preferred alternative, although it causes more side effects.^45-49^ Other laxatives, such as mineral oil, may be used as a second-line option if osmotic laxatives fail or are insufficient.^50^ Despite these alternatives, macrogol remains the most prescribed agent, as reflected in the study sample, which showed a low rate of non-prescription (7; 10.4%). Studies suggest that enemas, while useful for immediate relief, are not recommended for continuous therapy, which justifies their reduced use in the clinic (17; 25.4%).^9,44,51,52^ Mineral oil, although effective, is restricted due to risks such as aspiration and lipoid pneumonia as well as the potential for intestinal dependencewith prolonged use,^53- 56^ explaining its low prescription rate in the sample (7; 10.4%). 

 Although they do not have a prophylactic role,^ 32-36^ there is a recognized need for concurrent non-pharmacological measures for the effective management of functional constipation.^44^ In the present study, this therapeutic approach was recommended for just over half of the patients (38; 56.7%). This aligns with the literature, which points out that low water and fiber intake is associated with harder stools, worsening the condition, and is a critical component in managing chronic functional constipation.^57^ Lifestyle changes, such as increased physical activity and the establishment of a regular evacuation routine, were proposed for 35 (52.2%) individuals. However, no randomized studies have evaluated the effect of increased physical activity on childhood constipation, limiting evidence regarding its effectiveness.^44^

 Most children who received prescriptions used up to one medication to achieve complete symptom improvement, a stage reached by most patients (49; 73.1%). The number of medications required for the successful management of functional constipation can vary significantly based on individual responses to treatment and severity of the condition. For 60–90% of children, a single medication, typically macrogol, may be sufficient; however, some may not achieve satisfactory results and require additional therapies,^58^ as evidenced by eight patients in the present study who showed only partial improvement. Furthermore, 6 patients (9%) did not return for follow-up, and various factors contributed to this lack of follow-up, including socioeconomic barriers, lack of awareness among parents about the importance of follow-up, and logistical challenges such as transportation or scheduling issues.^59^ Without appropriate follow-up, healthcare providers may miss the opportunity to modify treatment plans or intensify care for those who do not respond adequately,^44,46 ,60,61^ resulting in chronic constipation and unsatisfactory long-term outcomes.^58^

 Although the analyzed medical records were comprehensive, some limitations should be acknowledged. The observational nature of this research, combined with its reliance on secondary data, may restrict the availability of information and prevent the inference of associations between variables. Additionally, the sample in this study, although relevant to the context of southern Santa Catarina, had limited national representativeness, which may have affected the generalizability of the results. Finally, factors such as lack of information on clinical progression and absence of follow-up data may affect the evaluation of the effectiveness of the proposed interventions. 

 This study provides an updated sociodemographic, clinical, and therapeutic profile of pediatric functional constipation in Brazil, addressing the scarcity of recent data in the national literature. With only one recent study available in 2021 and others dating back to the 2000s and the 2010s, the observations highlight distinct characteristics that may influence the understanding and management of the condition. Furthermore, by including a representative sample of the population attending a pediatric outpatient clinic, the results offer valuable insights into the development of more effective treatment and follow-up strategies tailored to the specific needs of this population. 

## CONCLUSION

 The analysis of 67 patients with functional constipation, representing 9.4% of the 710 pediatric gastroenterology outpatient visits at the PMC, revealed a predominantly female profile (36; 53.7%), with symptom onset before the age of 5 years and a mean age at consultation of 7.75 (± 3.96) years. The most commonly reported symptoms included abdominal pain (n = 52, 77.6%), hardened stools (n = 42, 62.7%), and fecal incontinence (n = 23, 34.3 %). Pharmacological treatment was characterized by frequent macrogol prescriptions (60, 89.6%), with most patients (49, 73.1%) achieving complete symptom relief. Non-pharmacological interventions such as dietary modifications were also implemented in more than half of the cases (38; 56.7%), reinforcing the importance of a multidisciplinary therapeutic approach. 

 For future research, a longitudinal approach is recommended to allow patient follow-up over time and to evaluate factors influencing treatment response, in addition to multicenter studies aimed at a more comprehensive understanding of functional constipation and its nuances. 
